# TiO_2_ nanotubes guided Pt nanorods growth via atomic layer deposition for mild photothermal antibacterial activity and enhanced fibroblast response

**DOI:** 10.3389/fbioe.2025.1672011

**Published:** 2025-09-09

**Authors:** Huan Liu, Wangle Zhang, Boya Xu, Dongxuan Cai, Haochen Wang, Xiaofeng Chang, Qin Zhou, Hao Feng, Wen Song, Zhe Li

**Affiliations:** ^1^ Key laboratory of Shaanxi Province for Craniofacial Precision Medicine Research, College of Stomatology, Xi’an Jiaotong University, Xi’an, China; ^2^ Laboratory of Material Surface Engineering and Nanofabrication, National Key Laboratory of Energetic Materials, State Key Laboratory of Fluorine and Nitrogen Chemicals, Xi’an Modern Chemistry Research Institute, Xi’an, China; ^3^ The 941 Hospital of the Joint Service Support Force of the People’s Liberation Army of China, Xining, China; ^4^ Department of stomatology, The 908th Hospital of the Chinese People’s Liberation Army Joint Logistic Support Force, Nanchang, China; ^5^ Department of Prosthodontics, School of Stomatology, State Key Laboratory of Oral & Maxillofacial Reconstruction and Regeneration, Shaanxi Key Laboratory of Stomatology, National Clinical Research Center for Oral Diseases, The Fourth Military Medical University, Xi’an, China; ^6^ Department of digital oral implantology and prosthodontics, College of Stomatology, Xi’an Jiaotong University, Xi’an, China

**Keywords:** dental implant, mild photothermal therapy, atomic layer deposition, antibacterial, fibroblast

## Abstract

**Introduction:**

Soft tissue integration (STI) and antibacterial capability are critical for the long-term success of dental implants. However, achieving both simultaneously remains a major clinical challenge. This study aims to address this challenge by developing a novel surface modification strategy that combines both robust antibacterial properties and favorable soft tissue responses for dental implants.

**Methods:**

A novel nanostructured coating was fabricated by embedding hollow platinum nanorods (PtNRs) into titania nanotubes (TNTs) using atomic layer deposition (ALD). The resulting PtNR@TNT platform was characterized to confirm its stable architecture and dual biofunctionality. Structural characterizations were conducted to verify that PtNRs were uniformly deposited along the inner walls of TNTs without compromising the nanotopography or hydrophilicity. Long-term in vitro degradation studies were performed to assess Pt retention and release. The antibacterial performance of the PtNR@TNT platform was evaluated under mild near-infrared (NIR) irradiation (0.5 W cm-2, 42 °C–45 °C) against S. aureus and *P. gingivalis*. Furthermore, human gingival fibroblasts (HGFs) were cultured on PtNR@TNT surfaces to study cell adhesion, proliferation, and migration. qPCR analysis was used to assess gene expression related to soft tissue responses.

**Results:**

The PtNR@TNT platform exhibited a stable structure with PtNRs uniformly distributed along the inner walls of TNTs, preserving the nanotopography and hydrophilicity. Long-term degradation studies demonstrated sustained Pt retention and minimal release of platinum (<50 μg L-1 over 12 weeks), indicating excellent safety. Under NIR irradiation, PtNR@TNT showed superior antibacterial activity, achieving over 99% bacterial reduction against *S. aureus* and *P. gingivalis* through a combination of photothermal and photodynamic effects. Additionally, HGFs demonstrated enhanced adhesion, proliferation, and migration on PtNR@TNT surfaces, with further promotion observed after NIR exposure. qPCR analysis revealed that NIR exposure upregulated the expression of key genes involved in soft tissue healing, including COL-1, FAK, ITGβ1, and VCL.

**Discussion:**

The findings from this study suggest that the PtNR@TNT platform offers a promising surface modification strategy for dental implants. It combines robust antibacterial efficacy with favorable soft tissue responses under clinically safe photothermal conditions. The dual biofunctionalities, including enhanced antibacterial performance and improved soft tissue integration, make the PtNR@TNT platform a promising candidate for advancing dental implant technology.

## Graphical Abstract

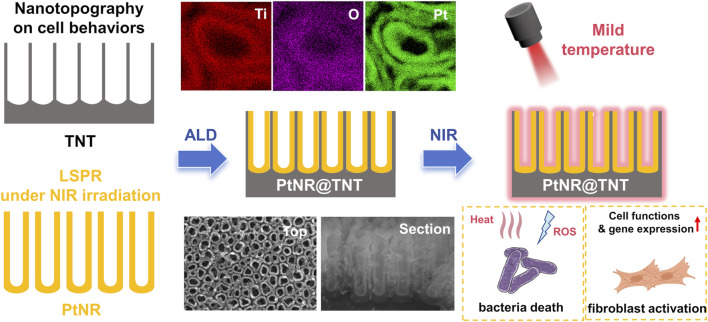

## 1 Introduction

Soft tissue integration (STI) around the emergence profile of dental implants is essential for establishing a biological barrier against pathogenic invasion and ensuring long-term implant stability (Chen et al., 2023). However, compared with natural dentition, peri-implant soft-tissue attachment is structurally weaker and more susceptible to microbial colonization, which may induce peri-implant mucositis and peri-implantitis, with reported incidence rates of 40%–65% and 20%–47%, respectively (Berglundh et al., 2018; Dixon and London, 2019; Tang et al., 2023). Therefore, strategies to promote rapid STI while simultaneously preventing bacterial infection at the transmucosal region are of great clinical importance. Among various approaches, nanotopography has been shown to enhance protein adsorption, cell adhesion, and hydrophilicity (Rivera-Chacon et al., 2013; Bachhuka et al., 2017; Cuahtecontzi Delint et al., 2024). Titania nanotubes (TNTs) are the among the most extensively studied nanostructures. Our group has dedicated substantial efforts to investigating the effects and mechanisms of TNT in osseointegration, with or without additional modifications (He et al., 2020; He et al., 2022; Wang et al., 2023a; Du et al., 2025). Nevertheless, achieving predictable antibacterial functionality while maintaining favorable soft tissue responses remains a key challenge.

Near-infrared (NIR)-mediated photothermal therapy (PTT) has emerged as a promising non-pharmacological strategy for precision antibacterial treatment (Wang et al., 2023b). Numerous studies have confirmed that noble metal nanorods (gold, silver, platinum) exhibit efficient and tunable photothermal effects via localized surface plasmon resonance (LSPR) (Hu et al., 2015; Taylor et al., 2022; Marhaba, 2025), particularly in hollow structure (Gan et al., 2022). However, a major limitation of PTT is its potential to damage surrounding healthy tissues. This risk becomes more pronounced under high-power irradiation (>1 W cm^-2^), which can induce local hyperthermia exceeding 50 °C (Wang et al., 2023b). To address this concern, mild photothermal therapy (mPTT) with a temperature range from 42 °C to 45 °C (Wang et al., 2022a), has gained increasing attention in recent years. Previous studies have demonstrated that the lower temperature range induces irreversible damage to heat-sensitive bacteria, whereas normal oral mucosa has been reported to tolerate such thermal exposure (Yi et al., 2021; Luo et al., 2023). Nonetheless, the antibacterial efficacy of mPTT diminishes as the thermal energy is reduced, presenting a challenge for clinical translation.

To enhance the antibacterial efficacy of mPTT, integrating multimodal strategies to achieve synergistic effects has shown great promise (Mei et al., 2024). Platinum (Pt) nanomaterials are well-recognized for their photothermal (Abed et al., 2022) and photodynamic responsiveness (Tomah et al., 2025). These properties theoretically enable Pt to enhance mPTT by synergistically inducing local hyperthermia and generating reactive oxygen species (ROS) to kill bacteria. The use of Pt to enhance photodynamic activity has been extensively studied (Wang et al., 2024; Tomah et al., 2025), including Pt-doped titania nanotubes (Vijayan et al., 2010). However, their application in antibacterial surface modification of oral implants via photodynamic therapy (PDT) has been rarely reported. This may be due to the difficulty in maintaining long-term surface stability of Pt, which contrasts sharply with tumor therapy strategies that rely on the rapid diffusion and cellular uptake of Pt-containing PDT systems. Therefore, it remains unclear how to stably incorporate Pt into the TNT array without disrupting its nanotopography, and whether this integration can concurrently enable effective mPTT-based antibacterial performance.

In this work, we report an interesting nanostructure in which hollow Pt nanorods (PtNRs) are firmly embedded into the TNT array (PtNR@TNT) via atomic layer deposition (ALD) technique, which is an atomic-level and controllable coating technique, capable of depositing substances into narrow spaces in a bottom-up manner (Zhang et al., 2022). Notably, TNT array not only serves as a confined template for PtNRs growth but also provides physically constraints for their stabilization (at least 3 months). *In vitro* experiments demonstrated that the topography-mediated enhancements of cell adhesion and proliferation conferred by TNTs were retained after PtNRs deposition. Simultaneously, PtNR@TNT exhibits dual photothermal/photodynamic effects, achieving effective antibacterial activity at a mild temperature (<45 °C) under 808 nm NIR irradiation without adversely affecting cell behaviors.

## 2 Materials and methods

### 2.1 Sample preparations

Pure titanium plates (1 mm thick, 15 mm in diameter; Baoji Titanium Industry Co., Ltd., China) were polished sequentially with 400 # to 3,000 # waterproof SiC sandpapers to obtain mirror-like titanium surfaces (Ti), then ultrasonically cleaned for 15 min in acetone, ethanol, and deionized water. TNT samples were fabricated by anodizing in 40 wt% hydrofluoric acid (Tianjin Fuyu Chemical Co, Ltd, China) at 20 V for 1 h. PtNRs were deposited onto TNT via ALD using an FH-2 ALD system (MCIR FH-2 ALD, Xi’an Modern Chemistry Research Institute, China) according previous study (Goulas and Van Ommen, 2013). Briefly, the TNT substrate was placed on a copper container in the ALD reactor. Trimethyl (methylcyclopentadienyl) platinum (IV) (MeCpPtMe_3_, Shanghai Yuanxiang Chemical Co., Ltd., China) and ozone (O_3_ produced using an ozone generator from ultrapure O_2_ (99.999%, Xi’an Weiguang Gas Co., Ltd., China)) served as the two precursors. ALD of Pt was performed at 150 °C, and each deposition cycle was comprised of: (1) 7 s MeCpPtMe_3_ exposure with a partial pressure of 8 Pa; (2) 20 s N_2_ purge (100 mL/min); (3) 20 s O_3_ exposure (80 mL/min); and (4) 20 s N_2_ purge (100 mL/min). The above cycle was repeated 50 times to achieve hollow PtNR deposition. All samples were stored in a vacuum chamber, and *in vitro* experiments were performed in 24-well plates (Corning Inc., United States), which matched the titanium discs.

### 2.2 Surface characterizations

The nanotopography was observed using FE-SEM (S-4800; Hitachi, Japan) and FE-TEM (JEM-ARM200F; JEOL, Japan). Surface roughness (Sq, 1 μm × 1 μm) was assessed by AFM (Shimadzu, Tokyo, Japan). Water contact angles were measured with a contact angle meter (DSA100S; Krüss GmbH, Germany). Surface composition and elemental mapping were performed using XPS (ESCALAB Xi+; Thermo Fisher Scientific, United States) and EDS (JED-2300T, JEOL, Japan). TNT and PtNR@TNT diameters were calculated from six representative SEM images using ImageJ, with Gaussian distribution fitted in GraphPad Prism 8.0 (*R* ^2^ represents the adjusted *R* ^2^ value).

### 2.3 *In vitro* degradation

All samples were immersed in 1 mL PBS (Boster; Wuhan, China) at room temperature. To mimic clinical conditions, NIR irradiation was applied during immersion—10 min/day (0.5 W cm^-2^), 3 days/week, for 12 weeks. At each interval, extraction solutions were fully collected, replaced with fresh PBS, and analyzed for Pt release using ICP-MS (NexION 350D, PerkinElmer Inc., United States). Extracts were also sterile stored for subsequent cell experiments. Surface morphology changes were monitored by SEM.

### 2.4 Photothermal performances

The absorption spectra were recorded using a UV–Vis–NIR spectrophotometer (Lambda 950, PerkinElmer, United States). To simulate physiological conditions, samples were immersed in 1 mL PBS and irradiated with an 808 nm NIR laser (0.5 W cm^-2^, BOT, China) for 10 min. The sample with the best photothermal response was further tested at varying power densities to determine optimal mPTT parameters. Photothermal stability was assessed via five laser on/off cycles. Temperatures were monitored using an infrared thermal camera (Fortric 628c; Fortric, China). Optimal NIR settings were selected for subsequent mPTT experiments. The calculation of photothermal conversion efficiency was performed based on the formula reported in our previous studies (Wang et al., 2022b).

### 2.5 Bacterial culture

*S. aureus* and *P. gingivalis* were selected as model bacteria. Frozen *S. aureus* was aerobically grown on Luria-Bertani (LB) agar for 24 h, then amplified in LB broth for 6 h. *P. gingivalis* was anaerobically cultured on Columbia blood agar for 5–7 days and expanded in brain heart infusion (BHI) broth. Before inoculation, bacterial densities were standardized using a McFarland turbidity meter. All media were supplied by Haibo Biotechnology Co., Ltd. (Qingdao, China).

### 2.6 Antibacterial experiment

1 mL suspension of *S. aureus* (5 × 10^6^ CFU/mL) or *P. gingivalis* (1 × 10^7^ CFU/mL) was added to TNT or PtNR@TNT samples and incubated for 12 h or 48 h, respectively. After NIR irradiation (0.5 W cm^-2^, 10 min), bacterial suspensions were collected. A 100 μL aliquot of each dilution was plated for CFU counting using high-resolution images. Supernatants were collected to measure protein and lactate dehydrogenase (LDH) leakages using Pierce™ BCA Protein Assay Kits (ThermoFisher Scientific, United States) and LDH Assay Kits (Abcam, United States). Bacterial survival rate was calculated using the formula:

$$\text{Bacterial }\text{survival }\text{rate }\left(\%\right)=A/B\times 100\;\%$$

A represents the CFU of the experimental groups, including TNT/NIR-, TNT/NIR+, PtNR@TNT/NIR-, and PtNR@TNT/NIR+, B represents control group (Ti/NIR-).

### 2.7 Bacterial morphology, viability, and biofilm assessment

After incubation and NIR treatment (0.5 W cm^-2^, 10 min), samples were rinsed with PBS. Bacterial morphology was assessed by SEM. For live/dead staining, samples were treated with DMAO/PI (BestBio, China) for 30 min and observed under a fluorescence microscope (CKX53; Olympus, Japan). As for biofilm analysis, *S. aureus* (1 × 10^7^ CFU/mL) and *P. gingivalis* (1 × 10^8^ CFU/mL) were co-cultured with samples for 48 h and 72 h, respectively. After NIR irradiation for 10min, samples were sonicated in PBS and the adherent bacteria was collected and resuspended in fresh medium. Biofilms were methanol-fixed, stained with 0.1% crystal violet, eluted with 33% acetic acid, and absorbance at 590 nm was measured using a microplate reader (Thermo Fisher Scientific, United States). Relative biofilm biomass (D) was calculated using the formula:

$$\text{Relative }\text{biofilm }\text{biomass }\left(\%\right)=C/D\times 100\;\%$$

C represents the OD_590_ value of each experimental group, including TNT/NIR-, TNT/NIR+, PtNR@TNT/NIR-, and PtNR@TNT/NIR+, D represents the OD_590_ value of control group (Ti/NIR-).

### 2.8 Detection of reactive oxygen species

*S. aureus* suspensions (1 mL, 5 × 10^6^ CFU/mL) were incubated with each sample for 12 h. After mPTT treatment, dichlorodihydrofluorescein diacetate (DCFH-DA) probe was added and incubated for 30 min to detect reactive oxygen species (ROS). All samples were then washed twice with PBS and *S. aureus* were collected and resuspended in equal volumes of PBS. The suspensions were transferred to a black 96-well plate, and fluorescence was measured at 488/525 nm (excitation/emission).

### 2.9 Cell culture

Primary human gingival fibroblasts (HGFs) were isolated from healthy human gingival tissue collected during the second-stage dental implant surgery, with approval from the Biomedical Ethics Committee of Health Science Center of Xi’an Jiaotong University (2025-XJKQIEC-KY-QT-0003–003). Tissues were digested in collagenase and cultured in α-MEM (Gibco, United States) containing 10% fetal bovine serum (FBS; PAN, Germany) and 1% penicillin-streptomycin (Solarbio, China). HGFs were identified by immunofluorescence staining (Supplementary Figure S1), and cells at passages 2 to 5 (P2-P5) were used for subsequent *in vitro* experiments.

### 2.10 Live/dead staining

HGFs were seeded onto each sample (50,000 cells/well) and incubated for 12 h. Samples were then exposed to NIR irradiation (0.5 W cm^-2^) for 10 min, followed by another 12 h incubation. Live/dead staining was performed using a Live/Dead Cell Stain Kit (Beyotime, China), and samples were observed by fluorescence microscopy.

### 2.11 Cell morphology and immunofluorescence staining

HGFs were seeded onto each sample (40,000 cells/well) and incubated for 12 h, followed by NIR irradiation (0.5 W cm^-2^, 10 min) and re-incubation for another 12 h. For SEM observations, samples were fixed and dehydrated through a graded ethanol series. For cytoskeletal analysis, cells were stained with DAPI (Beyotime, China) for 10 min and Alexa Fluor 488 phalloidin (Invitrogen, United States) for 1 h, then imaged using confocal laser scanning microscopy (CLSM; FV3000; Olympus, Japan). ImageJ software (NIH) was used for quantification.

### 2.12 Cell migration

Cell migration was evaluated using a scratch assay. Briefly, HGFs were seeded on all samples in 24-well plates equipped with silicon inserts (50,000 cells/well). NIR irradiation was applied after 12 h of pre-incubation, and HGFs were grown for another 12 h, then all samples were fixed for subsequent staining and CLSM observation.

### 2.13 Cell viability

After 24 h of pre-incubation, the cell viability was assessed using Cell Counting Kit-8 (CCK-8; Boster, China) at 0 h, 1 day, 3 days, and 5 days post-mPTT (0.5 W cm^-2^, 10 min). Then HGFs were incubated for another 1 h in fresh medium containing 10% CCK-8, 100 μL of supernatant was collected and transferred to 96-well plates (Corning Inc., United States), and the absorbance at 450 nm was measured with a microplate reader (Multiskan FC; Thermo Fisher Scientific, United States).

### 2.14 qRT-PCR analysis

To investigate the effects of mPTT applied at different times on gene expression, HGFs were simultaneously seeded (2 × 10^5^ cells/well) onto TNT and PtNR@TNT samples. The irradiation (0.5 W cm^-2^, 10 min) was separately applied at three different time baselines — 24 h, 36 h, and 48 h post-seeding (the protocol was illustrated in Supplementary Figure S2). All samples were harvested at 48 h after cell seeding for subsequent qPCR analysis. In brief, the total RNA was isolated and then reverse-transcribed into cDNA using the PrimeScript RT reagent kit (TaKaRa, Japan). Quantitative real-time PCR (qRT-PCR) was carried out using the TB Green^®^ Premix Ex TaqTM II (TaKaRa, Japan). Amplification reactions were performed on a CFX96 real-time PCR system, and the fluorescence data were analyzed using the Bio-Rad CFX Manager software. Glyceraldehyde 3-phosphate dehydrogenase (GAPDH) served as the housekeeping gene, and the 2^−ΔΔCt^ method was employed to evaluate the relative gene expression levels, including collagen type 1 (COL-1), focal adhesion kinase (FAK), integrinβ1 (ITGβ1), vinculin (VCL). Gene expression differences were calculated accordingly, and the sequences of all primers used are provided in Supplementary Table S2.

### 2.15 Western blot

HGFs (2 × 10^5^ cells/well) were seeded onto TNT and PtNR@TNT surfaces. NIR irradiation (0.5 W cm^-2^, 10 min) was applied 24 h post-seeding. Cells were harvested at 48 h and lysed with RIPA buffer (Beyotime, China) containing protease/phosphatase inhibitors (CST). Protein concentrations were determined using a BCA kit (Beyotime, China). Equal protein extracts were boiled, separated by SDS–PAGE, and transferred onto PVDF membranes. Membranes were incubated with specific primary antibodies (Supplementary Table S2) and GAPDH served as the loading control. Bands were semi-quantitatively analyzed using Fiji software.

### 2.16 Statistical analysis

All experimental procedures were independently replicated a minimum of three times. Quantitative results are reported as mean ± standard deviation. The groups were compared by one-way ANOVA using GraphPad Prism 8.0.1 (GraphPad Software Inc., San Diego, CA, United States). Statistical significance was defined as *p* < 0.05.

## 3 Results

### 3.1 Specimen characterizations

**FIGURE 1:**
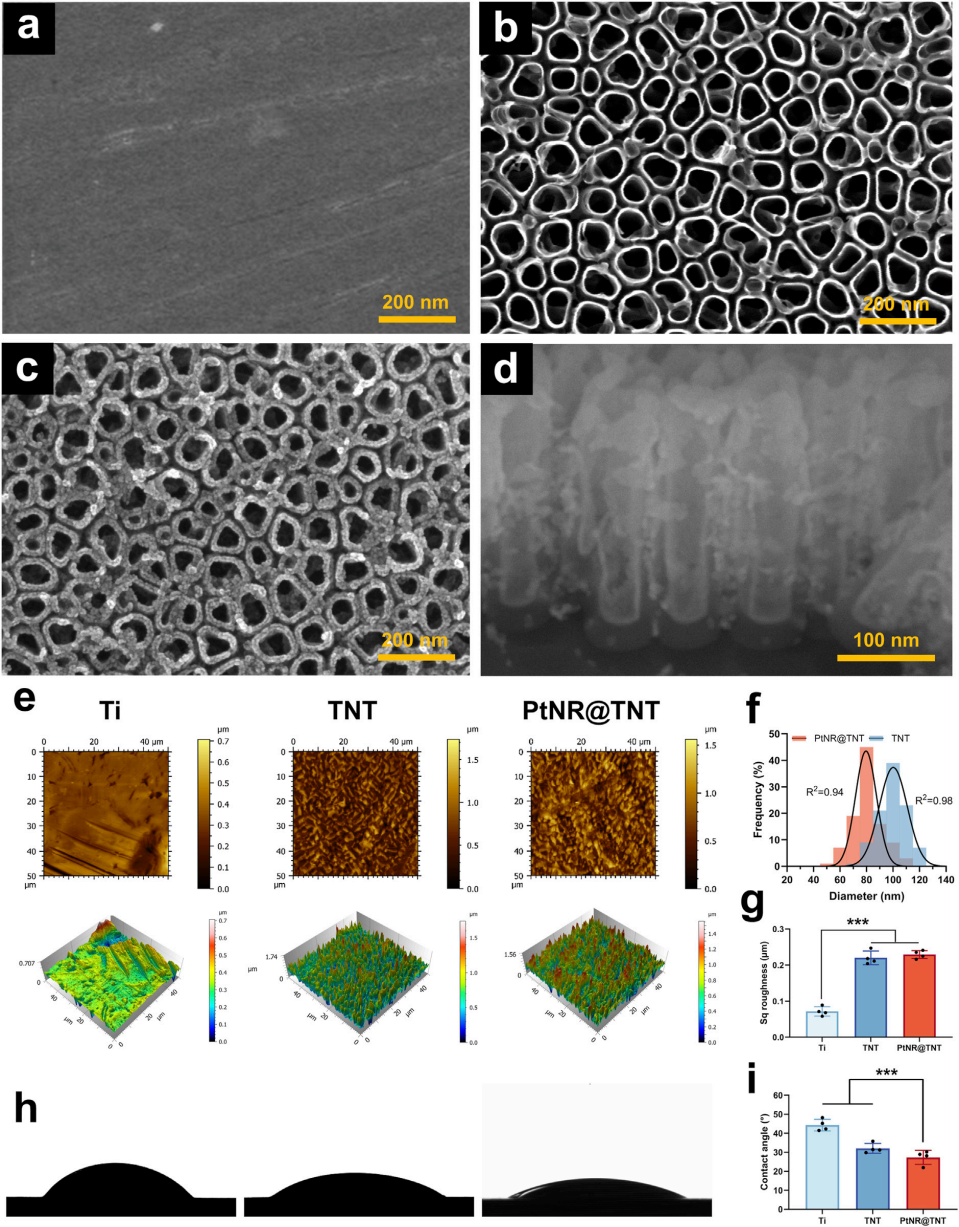
Specimen surface characterizations. FE-SEM observations of **(a)** Ti, **(b)** TNT, **(c)** PtNR@TNT in top view, together with **(d)** the cross-section view. **(e)** AFM observations of all samples. **(f)** Diameter changes of TNT and PtNR@TNT. **(g)** Sq roughness detected by AFM **(h)** Water contact angle images and **(i)** calculation. ^∗^ *p* < 0.05, ^∗∗^ *p* < 0.01, ^∗∗∗^ *p* < 0.001.

SEM observations showed that the polished surface of titanium substrate appeared well-ordered nanotube arrays after anodization (Figures 1a,b). After Pt ALD process, a uniform thickening of the nanotube walls was observed, while the porous nanotopography remained (Figure 1c). Cross-sectional observations further demonstrated that Pt, appearing brighter grayscales due to a higher atomic number, was uniformly deposited along the entire length of the nanotubes, forming a hollow Pt nanorod-in-TNT composite structure (PtNR@TNT, Figure 1d). The diameters of the nanotubes before and after PtNR loading were approximately 100 nm and 80 nm, respectively, both showing a normal distribution (Figure 1e). AFM observations revealed that the surface nanostructures of TNT and PtNR@TNT were basically the same, consistent with SEM results (Figure 1f). Sq roughness analysis showed an increased roughness of TNT (∼0.210 μm) compared to polished Ti (∼0.082 μm), while no significant difference was observed between TNT and PtNR@TNT (∼0.224 μm, Figure 1g). Water contact angle measurements showed that the contact angles of Ti, TNT, and PtNR@TNT were 43°, 30°, and 27°, respectively. Both TNT and PtNR@TNT exhibited significantly enhanced hydrophilicity compared to Ti, while no significant difference was observed between these two groups (Figures 1h,i).

**FIGURE 2:**
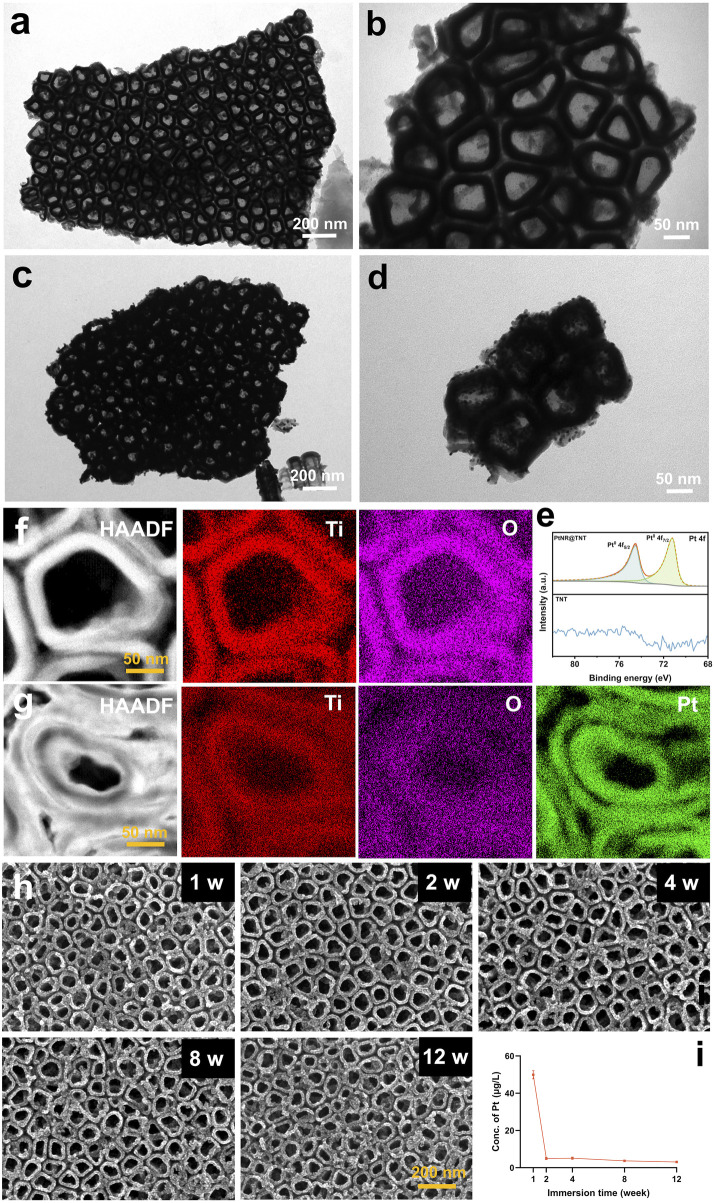
Characterizations and *In vitro* degradation. FE-TEM observation of **(a,b)** TNT and **(c,d)** PtNR@TNT. **(e)** XPS spectrum analysis and EDX mapping of **(f)** TNT and **(g)** PtNR@TNT with element analysis. **(h)** Surface observations in different immersing times and **(i)** related concentrations of released Pt.

FE-TEM observations revealed detailed nanostructures at high magnification. In particular, the inner and outer walls of the TNTs appeared clean and smooth before ALD (Figures 2a,b). Then, under the confinement of TNTs, Pt grew uniformly along the walls, forming hollow-like nanorod structures that were ultimately embedded within the TNT array. In addition, some Pt nanoparticles remained on the surface of the PtNRs due to the abrupt interruption of the ALD process (Figures 2c,d). Both XPS spectra (Figure 2e) and EDS mapping (Figures 2f,g) confirmed the successful loading of Pt onto TNTs, the results showed that titanium (Ti) and oxygen (O) elements composed the wall of the TNT, while and Pt was enriched adjacent to the wall.

### 3.2 *In vitro* degradation

To obtain long-term observation results, which are highly relevant for clinical applications, a 12-week evaluation was conducted. ICP-MS analysis showed a rapid initial release of Pt during the first week (∼48.8 μg L^-1^), followed by a significant decrease in degradation rate, reaching a stable level (∼4.3 μg L^-1^) after 2 weeks. Afterwards, no obvious changes were observed up to 12 weeks (Figure 2i). SEM observations were consistent with the ICP-MS findings, revealing no apparent nanostructural alterations of the PtNR@TNT throughout the 12-week period (Figure 2h).

### 3.3 Photothermal performances

**FIGURE 3:**
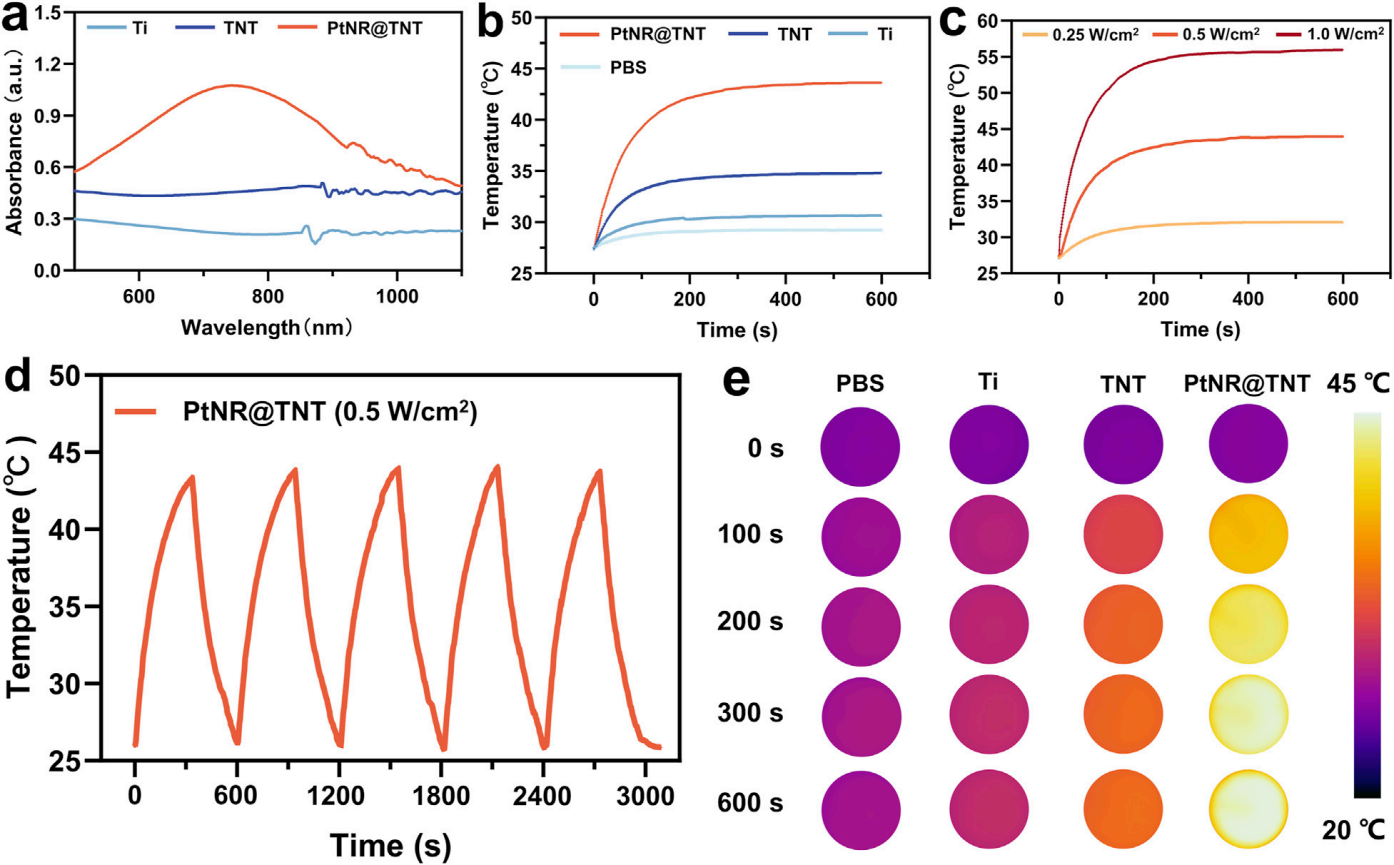
*In vitro* evaluations of the photothermal performances of all samples. **(a)** Absorption spectrum. The irradiation time-temperature curves for different **(b)** substrates, **(c)** power densities, and **(d)** cycles, together with **(e)** the images recorded by infrared thermal camera.

Visible-near infrared (VIS–NIR) absorbance spectra of the samples showed that Ti exhibited typical metallic mirror-like reflectance with low and flat absorption across the spectrum. Compared to Ti group, TNT showed increased absorbance, while PtNR@TNT demonstrated further enhanced absorption in the near-infrared I region (700–900 nm, Figure 3a). Under simulated physiological conditions, all samples reached thermal equilibrium at 200 s, with final temperatures of 30.3 °C (Ti), 34.2 °C (TNT), and 42.1 °C (PtNR@TNT), respectively. Only PtNR@TNT achieved the threshold temperature required for mPTT under 0.5 W cm^-2^ irradiation (Figure 3b). For PtNR@TNT, the temperatures at 200 s under different power densities were 31.6 °C (0.25 W cm^-2^), 42.5 °C (0.5 W cm^-2^), and 54.4 °C (1.0 W cm^-2^), indicating that only 0.5 W cm^-2^ meets the thermal requirement for mPTT (Figure 3c). After five cycles of NIR irradiation, PtNR@TNT consistently reached the target temperature (∼43 °C) without noticeable attenuation (Figure 3d). *In vitro* thermal imaging results were consistent with above findings (Figure 3e). The photothermal conversion efficiency of PtNR@TNT was 47.81%, which was markedly higher than that of TNT 22.56% (Supplementary Figure S3), indicating a significantly enhanced photothermal performance derived from the PtNR modification.

### 3.4 Antibacterial activity

**FIGURE 4:**
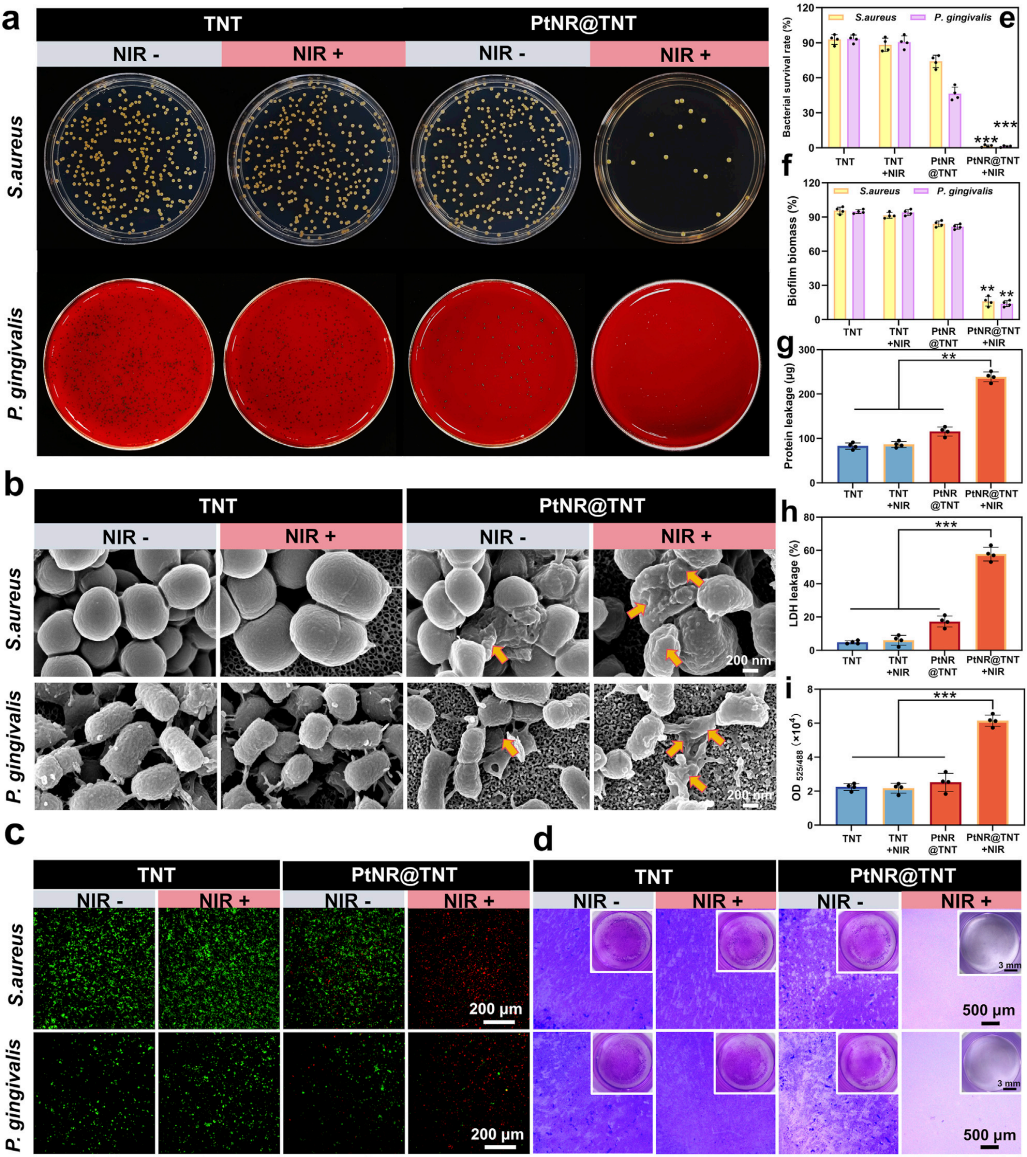
Antibacterial performance of PtNR@TNT-mediated mild PTT. **(a)** Photographs of bacterial colonies. **(b)** FE-SEM images of bacteria in different treatment groups for morphological damages detection (yellow arrow). **(c)** Live/dead fluorescence staining of bacteria after different treatments. **(d)** Biofilm formations in different treatment groups. The quantitative/semi-quantitative analysis of **(e)** bacterial survival rate, **(f)** biofilm biomass, **(g)** protein leakage, **(h)** LDH leakage and **(i)** ROS generation detected by DCFH-DA probe. ^∗^ *p* < 0.05, ^∗∗^ *p* < 0.01, ^∗∗∗^ *p* < 0.001.

Plate counting results visually showed the bacterial colonies of *S. aureus* and *P. gingivalis* were observed on the TNT surface without mPTT treatment, the survival rates of both bacteria were near 90% (Ti group served as control). PtNR@TNT exhibited moderate antibacterial effects even without irradiation, with survival rates of 83% (*S. aureus*) and 69% (*P. gingivalis*). After NIR exposure, these rates sharply decreased to 0.79% and 0.27%, respectively (Figures 4a,e). SEM observations revealed more pronounced bacterial death and membrane damage on the PtNR@TNT surface after mPTT treatment, while the bacteria grew normally on TNT surface regardless of NIR irradiation (Figure 4b). Live/dead staining showed numerous dead (red-fluorescent) bacteria on PtNR@TNT after NIR irradiation, whereas the TNT surface was predominantly covered by live (green-fluorescent) bacteria (Figure 4c). Biofilm visualization and quantification results were consistent with above findings (Figures 4d,f). Protein and LDH leakage assays further confirmed that NIR-treated PtNR@TNT induced greater intracellular content release, indicating membrane disruption (Figures 4g,h).

### 3.5 ROS detection

ROS detection based on DCFH-DA probe showed distinct differences among groups. As shown in Figure 4i, the OD values of TNT and PtNR@TNT without irradiation remained at low levels, indicating minimal ROS generation. After NIR irradiation, however, the PtNR@TNT group exhibited a marked increase in OD value, which was significantly higher than that of the TNT group under the same condition. This result demonstrates that PtNR@TNT under irradiation induces enhanced ROS production, suggesting a photodynamic contribution to bacterial inactivation.

### 3.6 Influences of mPTT on HGFs

**FIGURE 5:**
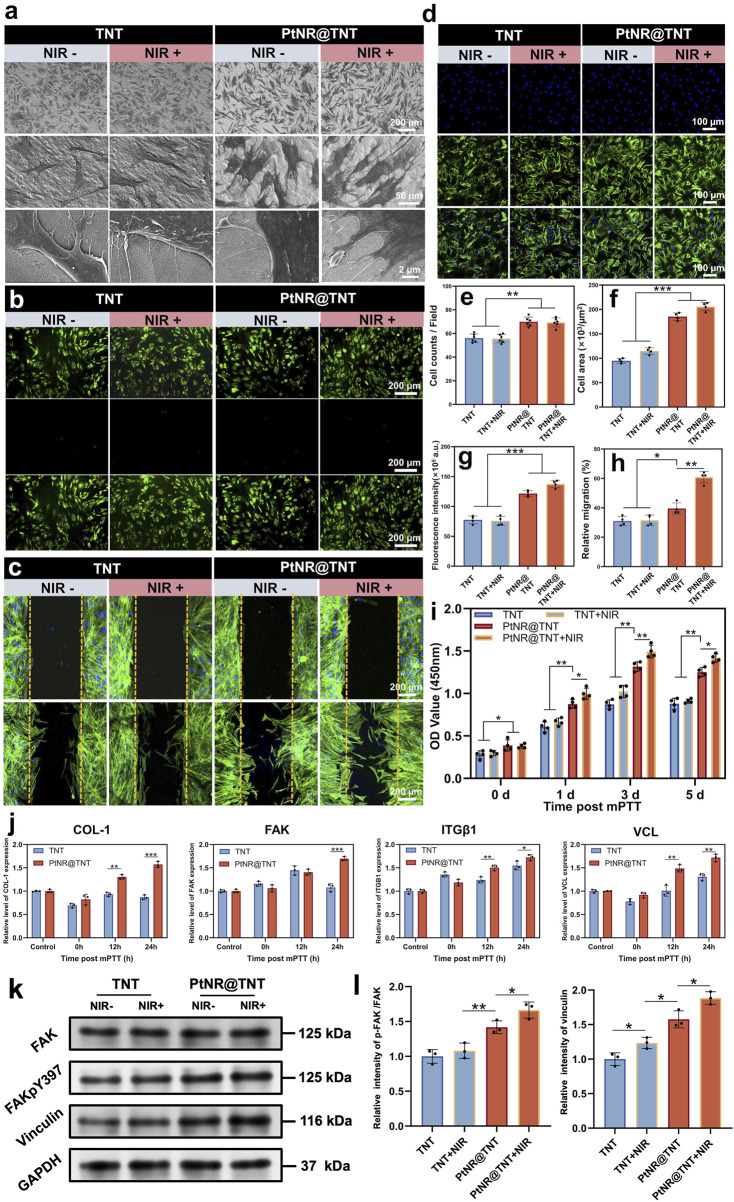
HGFs behaviors on PtNR@TNT with or without mild PTT. **(a)** FE-SEM images of cell morphology and their filopodia. Fluorescence staining of **(b)** live/dead cells, **(c)** migration, **(d)** attached cell and their cytoskeleton, together with the quantitative/semi-quantitative analysis of **(e)** attached cell amounts, **(f)** area, **(g)** fluorescence intensity of cytoskeleton, and **(h)** relative migration. **(i)** Cell viability measured by CCK-8. **(j)** Relative mRNA expression of four related genes, including COL-1, FAK, ITGβ1 and VCL. **(k)** the FAK, p-FAK, vinculin levels of HGFs on different groups, together with **(l)** semi-quantitative analysis. ^∗^ *p* < 0.05, ^∗∗^ *p* < 0.01, ^∗∗∗^ *p* < 0.001.

SEM observations of HGFs on the different surfaces showed that, cells on both TNT and PtNR@TNT exhibited normal morphology. However, compared to TNT, PtNR@TNT displayed more cell adhesion, greater cell spreading, and more filopodia formation (Figure 5a). Live/dead cell staining showed no cytotoxicity across the samples, with no significant cell damage observed post-NIR irradiation, confirming that the photothermal parameters selected for this experiment met the mPTT requirements (Figure 5b). *In vitro* scratch assay showed that HGFs migrated toward the center of the wells on all samples, but the cell migration on PtNR@TNT was significantly enhanced than that on TNT. NIR irradiation further enhanced the migration behavior, and quantitative analysis was consistent with CLSM results (Figures 5c,h). Immunofluorescence staining and fluorescence analysis further demonstrated a significant increase in both cell adhesion and cytoskeleton on PtNR@TNT surfaces, with no significant differences observed between NIR- and NIR + groups (*p* > 0.05, Figures 5d–g). CCK-8 results confirmed that cell viability of HGFs on all materials generally increased, with the highest viability observed in the PtNR@TNT + NIR group, followed by PtNR@TNT, and the lowest in the TNT and TNT + NIR groups (Figure 5i). qPCR results showed that 12 h post-mPTT treatment upregulated the expression of COL-1, ITGβ1, and VCL genes on PtNR@TNT surfaces, whereas FAK expression required 24 h post-mPTT treatment. No significant changes in gene expression were observed on TNT surfaces (p > 0.05, Figure 5j). Western blot analysis revealed that PtNR@TNT significantly increased the expression of focal adhesion–related proteins compared to TNT. Specifically, the p-FAK/FAK ratio was markedly elevated on PtNR@TNT, and vinculin expression was also upregulated. These effects were further enhanced after NIR irradiation, indicating that PtNR@TNT promotes focal adhesion activation and stabilization, particularly under mPTT conditions (Figures 5k,l).

To assess the long-term effects of Pt release on HGFs, a 2-week extraction solution was used as a conditioned medium to repeat the cell adhesion (24 h) and viability assays. The results showed that even exposure to the cumulative Pt released over 2 weeks did not impair HGF growth, further confirming the excellent biocompatibility of PtNR@TNT (Supplementary Figure S4). The hemolysis test of all groups was also performed according to previous study (Qin et al., 2025). The results showed that there was almost no hemolysis reaction in TNT or PtNR@TNT groups, even after NIR irradiation (the detailed data was shown in Supplementary Figure S5).

## 4 Discussion

Accelerating soft tissue integration (STI) and maintaining antibacterial activity are two essential functions required at the transmucosal region of titanium abutments, both critical for the long-term success of implant restorations. In this study, we employed advanced ALD technique to deposit Pt within the bioactive titania nanotubes (TNT), a series of characterization results confirmed the successful incorporation of Pt with a thickness of ∼10 nm. The minimal amount of Pt did not significantly change the nanotopography, roughness, or hydrophilicity of TNT, meanwhile, it effectively avoided potential cytotoxicity due to excessive Pt release.

In this study, the cumulative Pt release after 12 weeks remained below 50 μg L^-1^, this release amount was nearly one order of magnitude lower than the previously reported cytotoxic threshold (μg mL^-1^) (Almarzoug et al., 2020; Gurunathan et al., 2020). Such a safety margin provides strong assurance for subsequent *in vitro* and *in vivo* applications. Notably, most of the Pt released within the first 2 weeks, which is likely attributable to loosely bound Pt nanoparticles generated from the abrupt interruption of the ALD process, which could further be supported by FE-TEM observations. This also indicates that simply loading Pt nanoparticles onto implant surfaces may pose potential safety risks. Degradation behavior is a critical parameter in evaluating implant coatings, however, previous studies have often overlooked this aspect, focusing predominantly on functional performance. In this study, considerable effort was devoted to monitoring the degradation of PtNR@TNT over an extended period (12 weeks). The results revealed no apparent morphological changes, indicating excellent structural stability. This stability may be attributed to three main factors: (1) ALD relies on chemisorption rather than physisorption, forming covalent or coordination bonds with the substrate (Karasulu et al., 2016); (2) it enables uniform deposition within high–aspect ratio nanotubes with minimal aggregation; and (3) the confined geometry of TNTs physically anchors PtNRs, limiting their exposure to the external environment.

*P. gingivalis* and *S. aureus* are the predominant pathogens associated with periodontitis and early peri-implant infections, respectively, and are commonly used to evaluate the anti-infective properties of dental materials (Chen et al., 2024). As designed, PtNR@TNT functions as a photothermal/photocatalytic dual-mode antibacterial platform that 99.21% of *S. aureus* (1 × 10^7^ CFU/mL) and 99.73% of *P. gingivalis* can be eliminated at mild temperature (<45 °C). PtNRs incorporation significantly enhanced the photothermal performance of TNTs, as it exhibits an enhanced absorption in the near-infrared I region (700–900 nm) that enables rapid heating and stable maintenance at 43 °C under low power density (0.5 W cm^-2^), which meets the thermal requirements of mPTT. Although increasing energy intensity or Pt content may further raise the temperature, excessive heating may compromise biosafety. Fortunately, PtNR@TNT enhanced the efficacy of mPTT, with both ROS generation and extracellular matrix disruption potentially contributing to the antibacterial outcome, as evidenced by DCFH-DA fluorescence and significant leakage of intracellular protein and LDH. A similar result was reported by Deng et al., who achieved a photothermal/photodynamic/chemo tri-mode therapy by integrating a zero-valent Pt core with a bi-valent Pt shell (Deng et al., 2019). Notably, PtNR@TNT exhibited mild antibacterial effect even in the absence of irradiation, with *P. gingivalis* showing greater susceptibility than *S. aureus*, possibly due to early-stage Pt release. However, considering the minimal Pt release amount (<60 μg L^-1^) and the predominant contribution of photothermal/photodynamic therapy, such chemical effects were not considered an inherent antibacterial property.

Rapid soft tissue integration (STI) and the establishment of a physical barrier against bacterial invasion are essential functions of the transmucosal region. In this study, HGFs exhibited significantly enhanced adhesion, migration, and proliferation on the PtNR@TNT surface without NIR irradiation. Considering the morphology, roughness, and hydrophilicity remained basically unchanged after ALD treatment, these improvements are likely attributed to the presence of Pt and the preservation of the bioactive nanotubular nanostructure. Turner et al. reported that the incorporation of Pt enhanced the adhesion and spreading of Swiss mouse 3T3 fibroblasts (Turner et al., 2004), which was consistent with our findings. The enhancement of early cell functions without NIR irradiation indicates the intrinsic bioactivity of PtNR@TNT, which is critical for achieving rapid STI under non-pathological conditions. After NIR irradiation for 10 min, PtNR@TNT exhibited no significant cytotoxicity, instead, it further enhanced HGFs migration and proliferation on its surface. This finding is consistent with the study by Fu et al., which demonstrated that mPTT treatment (42 °C) significantly enhanced the migration and proliferation of L929 rat cells (Fu et al., 2024). It has been reported that mild heat enhances membrane permeability without impairing viability, facilitating biomolecule uptake by cells (Wolkers et al., 2019; Qu et al., 2022). It may also modify substrate properties to moderate the cell-surface interactions (Ebara et al., 2004). Consistent with previous findings, our study also demonstrated that post-mPTT treatment for 24 h significantly upregulated genes associated with cell adhesion and migration (e.g., FAK, ITGβ1, and vinculin) on the PtNR@TNT surface, accompanied by increased cytoskeletal formation. Western blot results also confirmed a higher p-FAK/FAK ratio and elevated vinculin expression on PtNR@TNT compared with TNT, and this difference became more pronounced after NIR irradiation, supporting enhanced focal adhesion activation. However, only a limited number of focal adhesion–related proteins were examined, and the downstream signaling pathways remain to be clarified in future studies. In addition, COL-1 (a key gene related to collagen synthesis) was significantly upregulated after mPTT treatment, indicating that HGFs were in an active biosynthetic state on PtNR@TNT surface which is beneficial for STI achievement (Yang et al., 2020).

From a translational perspective, three issues merit attention. First, the conformal nature of ALD suggests that PtNR@TNT can be applied to the transmucosal regions of standard abutments without altering their macro-design, enabling seamless integration with existing implant systems. Second, while ALD is costlier than conventional surface treatments, the extremely low Pt loading combined with scalable batch processing indicates that clinical application may still be economically feasible. Third, given that implant components must undergo sterilization (e.g., autoclaving or gamma irradiation), it is essential to confirm that PtNR@TNT retains its structural integrity and functional activity under such conditions. Addressing these aspects will be helpful to advancing PtNR@TNT from laboratory feasibility to clinical reality.

This study also has several limitations. (1) This *in vitro* study primarily focused on material fabrication, parameter optimization, and preliminary functional evaluation, with no *in vivo* experiments involved; (2) The respective contributions and mechanisms of PTT and PDT of PtNR@TNT in antibacterial and cellular responses remain unclear and the specific ROS subtypes underlying the antibacterial effect of PtNR@TNT remain undefined, which will require future clarification using more selective probes. (3) The long-term degradation behavior of PtNR@TNT requires investigation. Therefore, further study should address these aspects to support clinical application.

## 5 Conclusion

In summary, we developed a novel Pt nanorod–embedded titania nanotube platform (PtNR@TNT) via atomic layer deposition, which integrates stable nanotopography, dual-mode photothermal/photodynamic antibacterial capability, and excellent biocompatibility. This structure effectively eradicates pathogenic bacteria under mild photothermal conditions (<45 °C) while promoting human gingival fibroblast adhesion, proliferation, and migration. The antibacterial and bioactive effects of PtNR@TNT highlight its potential as a multifunctional transmucosal modification for dental implants. Further *in vivo* studies are warranted to validate its long-term therapeutic efficacy and clinical applicability.

## Data Availability

The original contributions presented in the study are included in the article/Supplementary Material, further inquiries can be directed to the corresponding authors.
