# Supplementary material for: TiO2 nanotubes guided Pt nanorods growth via atomic layer deposition for mild photothermal antibacterial activity and enhanced fibroblast response
Source: Front Bioeng Biotechnol. 2025 Sep 9;13:1672011. doi: 10.3389/fbioe.2025.1672011 (PMC12454311; doi:10.3389/fbioe.2025.1672011)
Supplement: Supplementary file 1 [file Supplementaryfile1.docx]

**Supporting information**

**TiO_2_ Nanotubes Guided Pt Nanorods Growth via Atomic Layer Deposition for Mild Photothermal Antibacterial Activity and Enhanced Fibroblast Response**

*Huan Liu^1#^, Wangle Zhang^2#^, Boya Xu^5#^, Dongxuan Cai^6^, Haochen Wang^4^, Xiaofeng Chang^1^, Qin Zhou^1^, Hao Feng^2*^, Wen Song^4*^, Zhe Li^1,3*^*

1. Key laboratory of Shaanxi Province for Craniofacial Precision Medicine Research, College of Stomatology, Xi’an Jiaotong University, Xi’an, 710004, China

2. Laboratory of Material Surface Engineering and Nanofabrication, National Key Laboratory of Energetic Materials, and State Key Laboratory of Fluorine and Nitrogen Chemicals, Xi’an Modern Chemistry Research Institute, Xi’an, 710065, China

3. Department of digital oral implantology and prosthodontics, College of Stomatology, Xi’an Jiaotong University, Xi’an, 710004, China

4. State Key Laboratory of Oral & Maxillofacial Reconstruction and Regeneration, National Clinical Research Center for Oral Diseases , Shaanxi Key Laboratory of Stomatology , Department of Prosthodontics，School of Stomatology , The Fourth Military Medical University, Xi’an, 710032, China

5. The 941 Hospital of the Joint Service Support Force of the People's Liberation Army of China, Xining, 810000, China

6. Department of stomatology, The 908th Hospital of the Chinese People's Liberation Army Joint Logistic Support Force, Nanchang, 550025, China

^#^Equal contribution

*Corresponding authors

Email: fenghao98@hotmail.com (H. F.); songwen71@fmmu.edu.cn (W. S.) and drlizhe@126.com (Z. L)

**
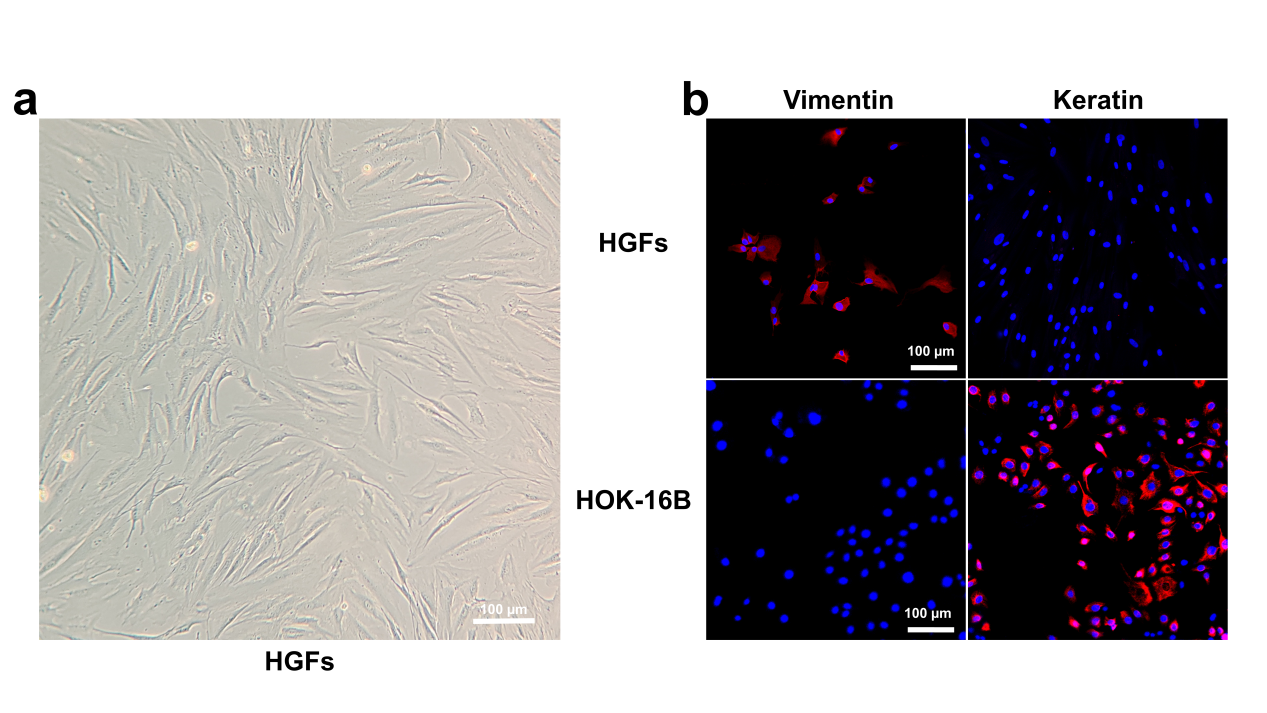
**

**Figure S1. HGFs culture and cell identification.** (a) Bright field image of HGFs. (b) Vimentin and Keratin expressions were visualized by immunofluorescence.


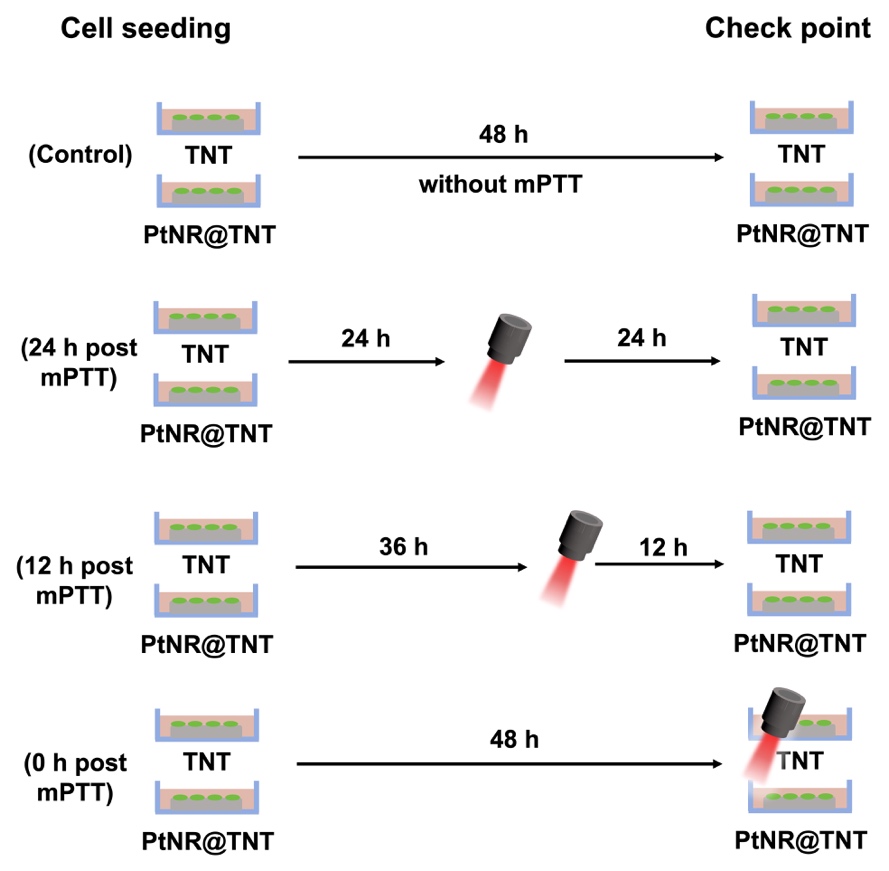


**Figure S2.** Experimental process and grouping diagram of qPCR analysis


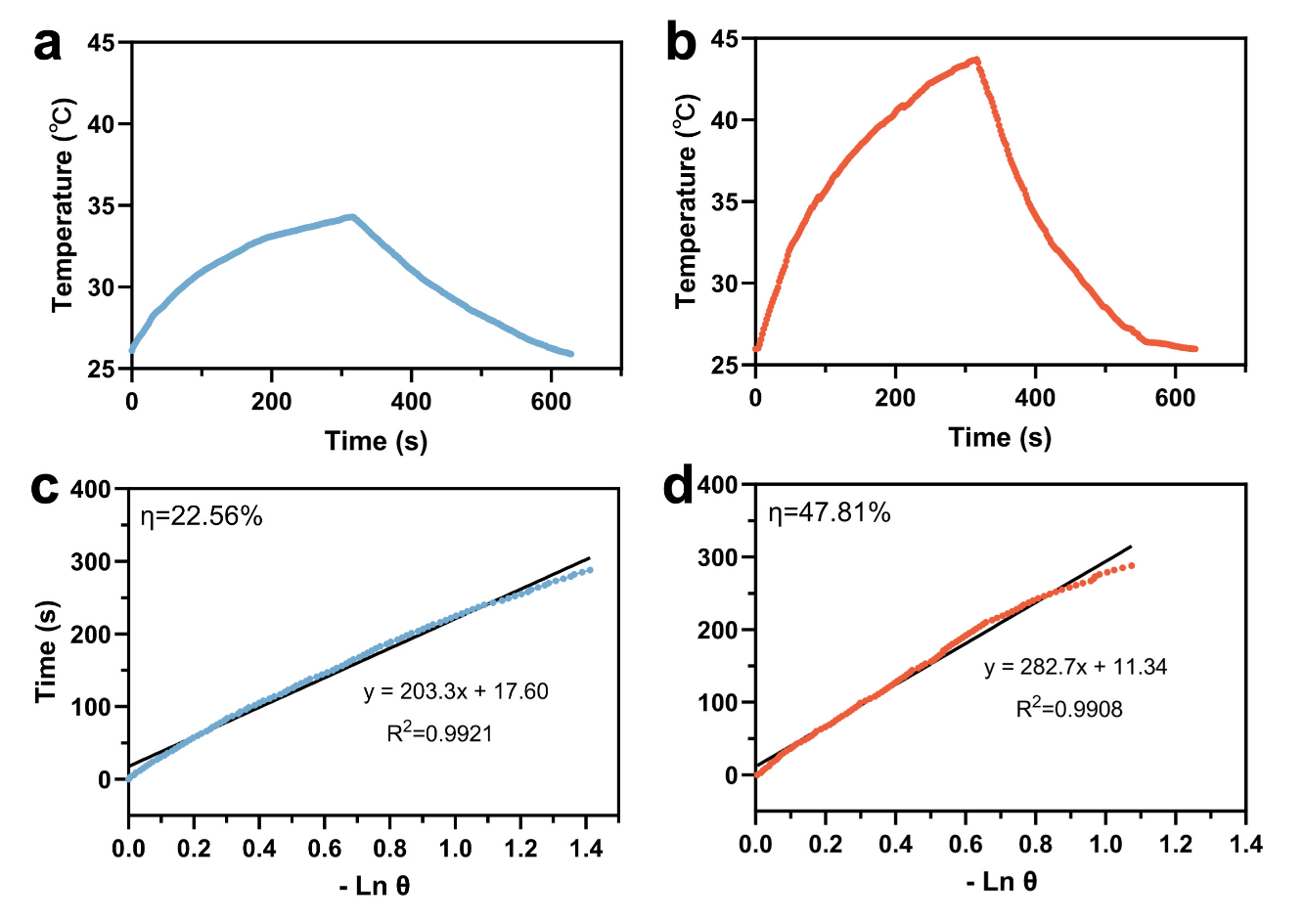


**Figure S3.** The temperature change curve of TNT (a) and PtNR@TNT (b) after irradiation and after turning off the laser; and the relationship between the cooling time and the negative natural logarithm of the temperature driving force for TNT (c) and PtNR@TNT (d). The photothermal conversion efficiency (η) of TNT and PtNR@TNT was calculated to be 22.56 % and 47.81 %, respectively.


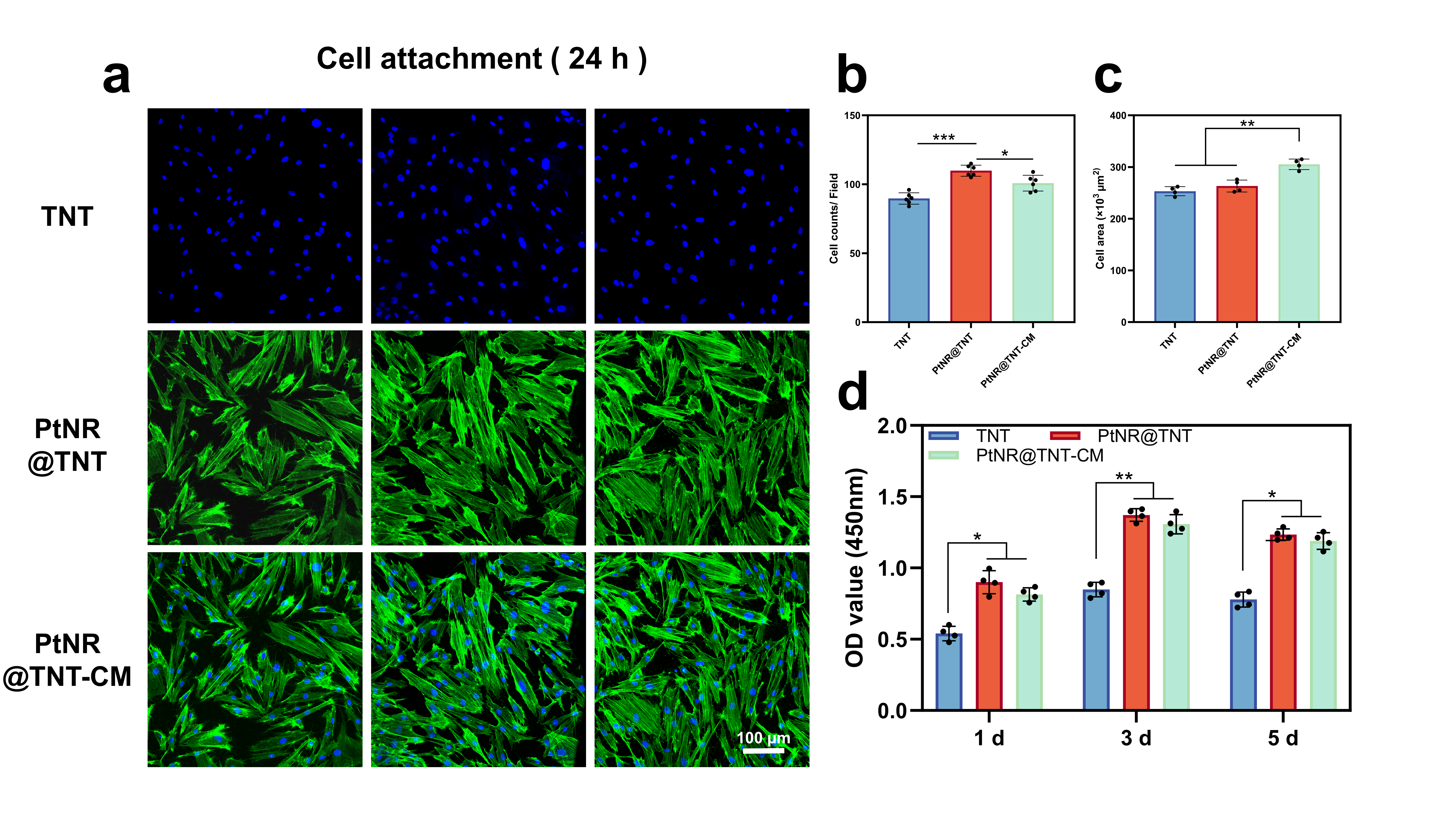


**Figure S4. Cell attachment and cell viability.** (a) Bright field image of HGFs. (B) Vimentin and Keratin expressions were visualized by immunofluorescence.


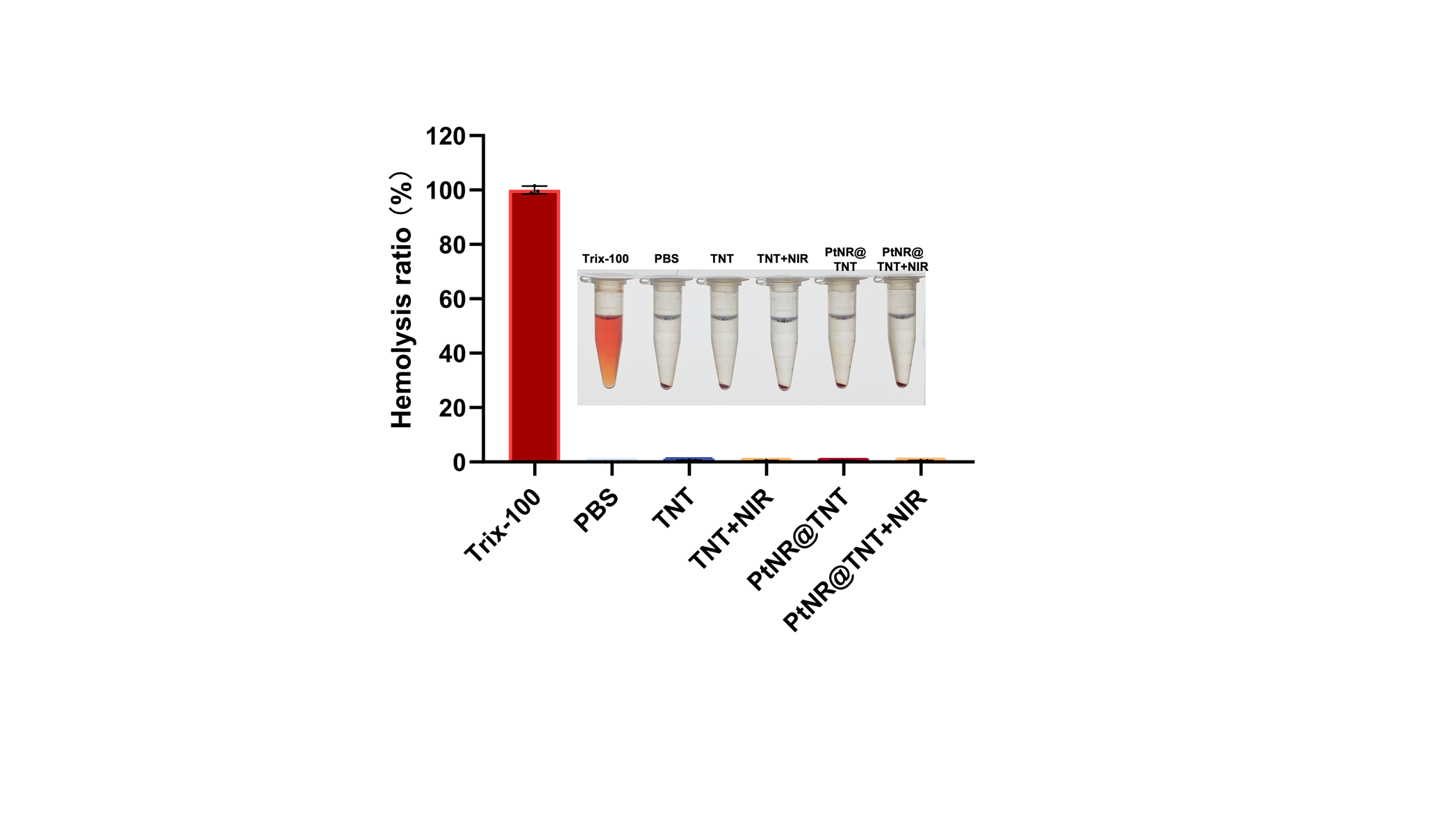


**Figure S5** **Hemolysis test *in vitro* for evaluation of blood compatibility of all samples.** Images showed the RBCs treated with Trix-100, PBS, TNT, TNT+NIR, PtNR@TNT, PtNR@TNT+NIR. Trix-100 group, and PBS group were positive control and negative control group, respectively; and the hemolysis ratio of each group was calculated according to the following equation: Hemolysis ratio (%) = (At − An) / (Ap − An) × 100%, where At is the absorbance of the experimental group, Ap is the absorbance of the positive control, and An is the absorbance of the negative control.


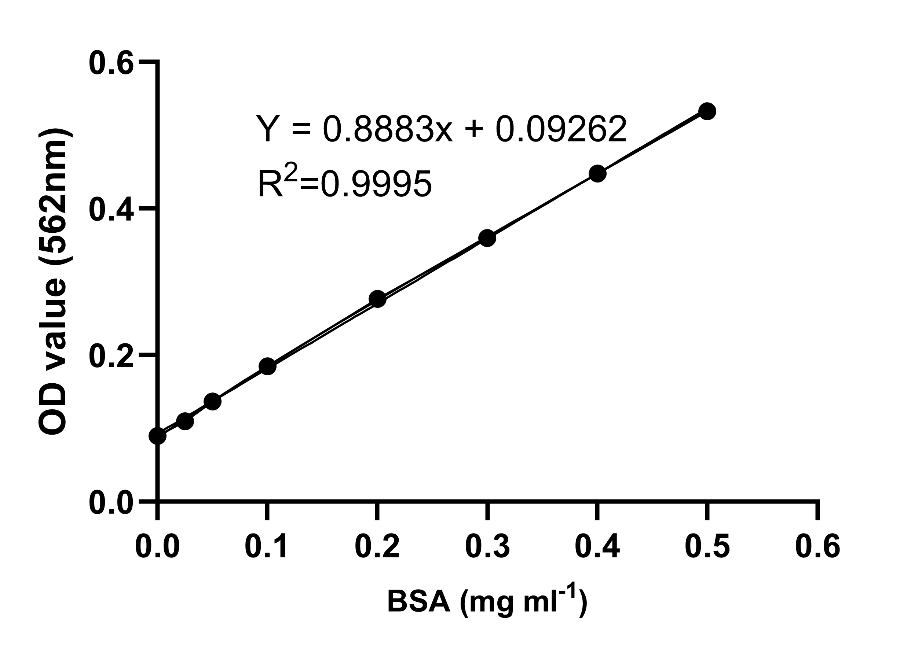


**Figure S6. A Representative standard curve for the protein assay.** Bovine serum albumin (BSA) was used as a standard and was diluted to the following concentrations: 0, 0.025, 0.05, 0.1, 0.2, 0.3, 0.4, and 0.5 mg/mL. Absorbance was measured at = 562 nm. Each data point represents an average of three absorbance readings per BSA concentration

**Table S1．**RT-qPCR primer sequence

| **GENE** | **Forward Primer Sequence (5'-3')** | **Reverse Primer Sequence (3'-5')** |
| --- | --- | --- |
| COL-1 | CCCAGCCACAAAGAGTCTACAT | GATTGGTGGGATGTCTTCGTCTT |
| FAK | CCTCCTACCCTCTACAGCCTTAT | CTGGACCTCGGACTGGGATAAC |
| ITGβ1 | TGATTGGCTGGAGGAATGTTACA | TTCTGGACAAGGTGAGCAATAGA |
| VCL | ACCAAGGCATAGAGGAAGCTTTAA | CAATGCTCTCTTCATGGCTTCAG |
| GAPDH | AGAAGGCTGGGGCTCAATTTG | AGGGGCCATCCACAGTCTTC |

COL-1，collagen I；FAK，focal adhesion kinase；ITGβ1，integrin β1；VCL，vinculin；GAPDH, glyceraldehyde-3-phosphate dehydrogenase.

**Table S2．**Antibodies used for WB

| **Antibody** | **Source** | **Identifier** |
| --- | --- | --- |
| FAK antibody | Cell Signaling Technology | Cat#3285 |
| FAKpY397 antibody | Invitrogen | Cat#44-624G |
| Vinculin | Cell Signaling Technology | Cat#4650 |
| GAPDH | CMCTag | Cat#AT0002 |

**Control**

**Incubation for 48 h**

**TNT**

**PtNR@TNT**

**TNT**

**PtNR@TNT**

**TNT**

**PtNR@TNT**

**Incubation for 48 h**

**TNT**

**PtNR@TNT**

**TNT**

**PtNR@TNT**

**Incubation for 24 h**

**TNT**

**PtNR@TNT**

**Incubation for 24 h**

**TNT**

**PtNR@TNT**

**TNT**

**PtNR@TNT**

**Incubation for 36 h**

**TNT**

**PtNR@TNT**

**Incubation for 12 h**

**TNT**

**PtNR@TNT**

**Cell seeding**

**Check point**

**24 h post mPTT**

**12 h post mPTT**

**0 h post mPTT**

**Control**

**Incubation for 48 h**

**TNT**

**PtNR@TNT**

**TNT**

**PtNR@TNT**

**TNT**

**PtNR@TNT**

**Incubation for 48 h**

**TNT**

**PtNR@TNT**

**TNT**

**PtNR@TNT**

**Incubation for 24 h**

**TNT**

**PtNR@TNT**

**Incubation for 24 h**

**TNT**

**PtNR@TNT**

**TNT**

**PtNR@TNT**

**Incubation for 36 h**

**TNT**

**PtNR@TNT**

**Incubation for 12 h**

**TNT**

**PtNR@TNT**

**Cell seeding**

**Check point**

**24 h post mPTT**

**12 h post mPTT**

**0 h post mPTT**
